# Study on Drag Reduction of Ti6Al4V with Different Shaped Microstructures via Femtosecond Laser Processing

**DOI:** 10.3390/ma19112183

**Published:** 2026-05-22

**Authors:** Mingwei Sun, Ying Wang, Jingying Li, Jinjun Wu

**Affiliations:** 1 China Academy of Machinery Science and Technology Group Co., Ltd., Beijing 100044, China; 2 Yanqi Lake Institute of Basic Manufacturing Technology Research Co., Ltd., Beijing 101400, China; wangy@pcmi.com.cn (Y.W.); jingying_cn@sohu.com (J.L.); 3 China Productivity Center for Machinery Co., Ltd., Beijing 100044, China; 4 Machinery Technology Development Co., Ltd., Beijing 100044, China; jinjun_wu@163.com

**Keywords:** titanium alloy, drag reduction microstructure, femtosecond laser processing, computational fluid dynamics

## Abstract

To enhance the aerodynamic performance of Ti6Al4V functional components, this paper systematically investigated the femtosecond laser processing technology for surface drag-reduction microstructures, aiming to fabricate high-performance microstructures. (1) V-shaped, U-shaped, and rectangular micro-grooves were designed based on the boundary layer theory, and their drag-reduction mechanisms were elucidated through CFD numerical simulations. The results indicate that the V-shaped groove achieves a peak drag-reduction rate of 13.1% at a dimensionless depth of *h^+^* = 15 and an aspect ratio of 1, primarily due to the formation of a low-velocity zone and the suppression of turbulent bursts by secondary vortices. (2) Through single-factor experiments, the influence laws of femtosecond laser process parameters on the V-shaped groove were explored. (3) Regression prediction models for groove dimensions were established using the Response Surface Methodology (RSM) to optimize the processing parameters. Under the optimized conditions, high-quality V-shaped groove arrays with a width of 55.9 μm and a depth of 55.5 μm were successfully fabricated on the Ti6Al4V surface, characterized by high consistency and a minimal heat-affected zone. This research provides an effective technical solution for the precision manufacturing of high-performance drag-reduction structures on titanium alloy surfaces.

## 1. Introduction

Ti6Al4V titanium alloys are regarded as cornerstone materials in the aerospace industry, particularly for aerodynamic functional components, owing to their superior strength-to-weight ratio, thermal stability, and environmental resilience. However, in high-velocity flow regimes, the intense skin friction drag manifested at the fluid-solid interface results in substantial parasitic energy losses and a marked diminution in propulsion efficiency. Consequently, developing effective surface-mediated drag-reduction strategies while preserving the intrinsic properties of titanium alloys has emerged as a pivotal challenge within the aerospace sector [1].

Currently, the aerodynamic layout of aircraft has become relatively mature. The research on drag reduction has gradually focused on regulating the characteristics of the fluid boundary layer through active or passive flow control technologies, thereby reducing drag. Among them, inspired by the structure of the shark skin’s shield scales, surface micro-grooves have become a research hotspot due to their advantages such as high structural stability, ease of processing, and no additional weight increase [2,3].

Regarding the drag-reduction effect of surface micro-grooves, numerous studies have been conducted by scholars both at home and abroad. Wang et al. [4] designed three types of groove structures: triangular, rectangular, and trapezoidal. Through numerical simulation and wind tunnel experiments, they confirmed that the trapezoidal groove could effectively control the additional pressure difference drag while reducing the wall shear stress. Liu et al. [5] used the third-generation Ω vortex identification method to compare the drag-reduction performance of V-shaped, blade-shaped, and arc-shaped grooves. They found that the blade-shaped groove had the highest drag-reduction rate of up to 18.2% due to the formation of a stable and fully developed near-wall vortex isolation layer. Chen et al. [6] used the laser ablation-chemical etching process to fabricate periodic semi-circular microstructures on the stainless steel surface. They achieved a drag-reduction rate of 29.83% in the micro-channels, providing an effective method for the fabrication of drag-reducing microstructures on metal surfaces. Bixler et al. [7] conducted systematic closed-channel flow experiments to establish the drag-reduction performance databases for blade-shaped and sawtooth-shaped micro-grooves. They found that the hydrophobic-modified blade-shaped groove had the highest drag-reduction rate of up to 34% in the flow, and proposed key design criteria such as h/s and t/s, providing theoretical basis for subsequent microstructure optimization design. Martin et al. [8] studied various continuous and segmented longitudinal rib-like geometric shapes and vortex structures of blades and found that the resistance of the continuous configuration was reduced by approximately 10%. Khader et al. [9] set up different depths and widths of microstructures on the turbine rotor surface and investigated the influence of microstructure shape and arrangement on the drag-reduction effect. The research results showed that the best drag-reduction effect was achieved when *h^+^* = 11.8, while the resistance increased when *h^+^* > 19.3.

Nevertheless, at present, the research on the drag-reducing microstructures of titanium alloy materials is relatively scarce, especially in terms of systematic design and process implementation under high flow velocity conditions. Meanwhile, titanium alloy is a typical difficult-to-machine material. Traditional mechanical processing is prone to generating burrs and having a large heat-affected zone, making it difficult to meet the processing accuracy requirements for micrometer-sized grooves. Femtosecond laser processing, as an ultra-short pulse processing technology, has advantages such as minimal heat-affected zone, high processing accuracy, and wide material adaptability. It shows great potential in the manufacturing of micro-nano structures on metal surfaces [10,11]. Existing studies have shown that femtosecond laser can be used for the processing of micro-pores and micro-grooves on the surface of titanium alloy [12]. Xu et al. [13] used laser micro-nano manufacturing technology to successfully create biomimetic sharkskin composite micro-nano structures on the surface of the hemispherical head-body model of an aircraft, achieving a drag-reduction rate of up to 10.3%. Wang et al. [14] and Cheng et al. [15] used femtosecond laser to fabricate micro-groove textures with different spacings on the surfaces of 304L stainless steel and hard alloy, and found that under appropriate spacings, it can effectively enhance the drag-reduction ability of the stainless steel surface. However, there is still a lack of systematic research on the optimization of process parameters and morphology control for specific drag-reducing microstructures.

Against this backdrop, this study employs Ti6Al4V titanium alloy as the substrate to systematically investigate three distinct drag-reduction micro-geometries: V-shaped, U-shaped, and rectangular grooves. Initially, the drag-reduction mechanisms are elucidated via Computational Fluid Dynamics (CFD) simulations, facilitating the identification of the optimal structural configuration and its requisite geometric parameters. Subsequently, single-factor experiments are conducted to discern the influence of laser power, number of scans, and scanning width on the resulting groove morphology, thereby establishing a preliminary processing window. Finally, a predictive regression model is developed using the Response Surface Methodology (RSM) to optimize the process parameters, enabling the fabrication and characterization of high-consistency V-shaped arrays. Accordingly, the remainder of this paper is organized as follows: Section 2 details the micro-structure design and simulation analysis; Section 3 describes the femtosecond laser ablation experiments and optimization framework; and Section 4 summarizes the key findings and provides concluding discussions.

## 2. Materials and Methods

Firstly, the drag-reduction structure is designed. The size interval of the drag-reduction structure is solved by using the boundary layer theory. Meanwhile, the existing microstructure shapes are selected in combination with the characteristics of laser processing, and simulation experiments with different shapes and size parameters are set. Then, based on the computational fluid dynamics method, a simulation method for the drag-reduction performance of grooved plates was established. On the basis of verifying the model accuracy, the drag-reduction effects of different microstructures were explored, and the influences of dimensionless depth and width-to-depth ratio on drag reduction were analyzed. Meanwhile, the drag-reduction mechanism of microstructures was studied from the perspectives of the “second vortex group theory” and the “protrusion height theory”.

### 2.1. Boundary Layer Theory

The boundary layer is a thin layer with a distinct flow velocity gradient formed by the viscous effect of a fluid near a stationary flat wall. It is defined as the area where the flow velocity gradually increases from zero velocity on the wall to 99% of the free flow velocity ($u_{0}$). According to Pratt’s boundary layer concept, if the fluid flow velocity $u\left(y\right)$ within the boundary layer is defined, and y is the distance from this point to the wall, then we have:

(1) $$u\left(\delta \right)=0.99u_{0}$$

where δ represents the boundary layer thickness and $u_{0}$ is the free flow velocity.

**Figure 1:**
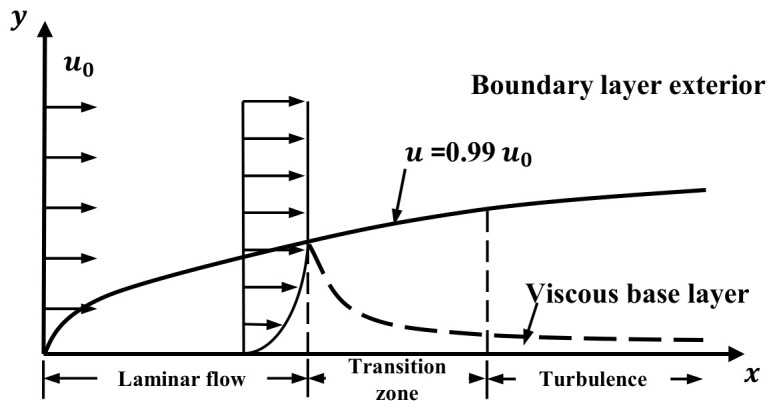
Plate flow-around model.

In the plate flow-around model shown in Figure 1, the fluid states along the flow direction are laminar flow, transition zone and turbulent flow in sequence, and the thickness of the boundary layer gradually increases along the flow direction [16].

**Figure 2:**
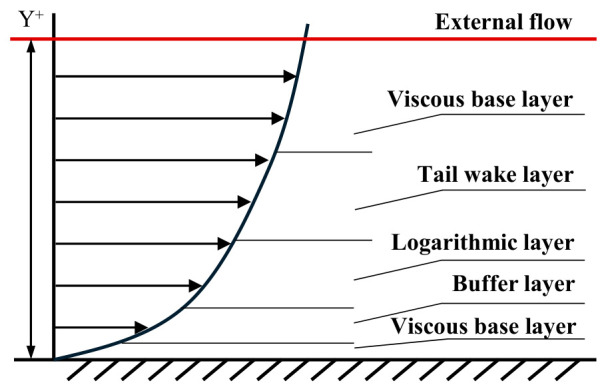
Schematic diagram of the distribution within the turbulent boundary layer.

In the turbulent region, it can be divided into five layers: the viscous bottom layer, the buffer layer, the logarithmic law layer, the wake law layer and the viscous top layer, as shown in Figure 2. The outside of this region is regarded as the outflow zone [17]. Introducing the dimensionless quantity y^+^ can represent the thickness of the boundary layer:

(2) $$y^{+}=\frac{yu_{\tau }}{v}=\frac{y}{v}\sqrt{\frac{\tau_{0}}{\rho }}$$

where y^+^ represents the dimensionless distance from the wall, y is the actual distance from the wall, $u_{\tau }$ is the wall shear velocity, v is the kinematic viscosity of the fluid, $\tau_{0}$ is the wall shear stress, and ρ is the fluid density.

When 0 ≤ y^+^ ≤ 5, this area is a viscous bottom layer and is mainly subjected to viscous shear stress. In the viscous bottom layer, the flow pattern of the fluid mainly exhibits characteristics similar to laminar flow, and the velocity distribution is approximately linear. When 5 ≤ y^+^ ≤ 30, this area serves as a buffer layer, mainly subjected to viscous shear stress, while turbulent shear stress also exists. When 30 ≤ y^+^ ≤ 100, this area is a logarithmic law layer, mainly subjected to turbulent shear stress, and there is a logarithmic relationship between the velocity and the wall height. When 100 ≤ y^+^ ≤ 0.4δ, this area is the wake law layer, and the flow state is completely turbulent. The relationship between the velocity and the wall height also shows a logarithmic relationship. When 0.4δ ≤ y^+^ ≤ δ, this region is the viscous top layer and the outermost layer of the boundary layer. The velocity distribution is very complex and is usually determined by experiments.

### 2.2. Drag-Reduction Microstructure Design

#### 2.2.1. Solution of Microstructure Size Range

Before limiting the range of microstructure depth, the dimensionless depth *h*^+^ of the microstructure is defined by referring to the definition method of boundary layer thickness:

(3) $$h^{+}=\frac{hu_{\tau }}{v}$$

(4) $$u_{\tau }=\sqrt{\frac{\tau_{0}}{\rho }}$$

(5) $$\tau_{0}=0.0225\rho u^{2}{\left(\frac{v}{u\delta }\right)}^{1/4}$$

where h represents the actual depth from the wall, $u_{\tau }$ is the shear velocity of the wall, v is the kinematic viscosity of the fluid, $\tau_{0}$ is the shear stress of the wall, ρ is the density of the fluid, and u is the flow velocity of the fluid.

The boundary layer thickness δ increases with the increase in the distance from the leading edge of the plate, and the relationship is expressed as follows:

(6) $$\delta =0.37\;\times \;{Re}_{x}^{-\frac{1}{5}}$$

The calculation formula for the Reynolds number ${Re}_{x}$ within the flow layer around the plate is as follows:

(7) $${Re}_{x}=\frac{ux}{v}$$

(8) μ=ρ·v

where x represents the characteristic length of the plate, and μ is the dynamic viscosity coefficient of the fluid. In this paper, the fluid is empty, and the characteristic parameters of air are shown in Table 1.

Substituting Equations (5)–(7) into Equation (4), we obtain:

(9) $$u_{\tau }=0.029\rho u^{2}{Re}_{x}^{-\frac{1}{5}}$$

Substituting Equation (9) into Equation (3), we get:

(10) $$h^{+}=0.172\frac{hu{Re}_{x}^{-\frac{1}{10}}}{v}$$

The formula for calculating the thickness of the viscous base layer is as follows:

(11) $$\varepsilon =\frac{5\mu }{u_{\tau }\rho }$$

According to the hydraulic roughness theory, when the depth of the microstructure is greater than the thickness of the viscous bottom layer, the microstructure has a drag-reduction effect. Moreover, the research results of Walsh’s team show that the drag-reduction effect is the best when *h^+^* < 30, and it is necessary to ensure that the width-to-depth ratio is within the range from 0.5 to 2. In summary, the equations for solving the microstructure size range are as follows:

(12) $$\left\{\begin{array}{c} h^{+}=0.172\frac{hu{Re}_{x}^{-\frac{1}{10}}}{v}<30\\ h>\varepsilon =\frac{5\mu }{u_{\tau }\rho }\\ 0.5\;h<s<2\;h \end{array}\right.$$

In this paper, the incoming flow velocity is set at 100 m/s. By substituting the characteristic parameters of the air into the given conditions in combination with the above formula, 18 µm < *h* < 108 µm can be obtained. The incoming velocity of 100 m/s is set according to typical aerospace working conditions. As a dimensionless parameter, *h^+^* is independent of Reynolds number. Thus, the optimal *h^+^* = 15 and aspect ratio = 1 obtained in this study can be generalized to different Reynolds number environments based on boundary layer scaling.

#### 2.2.2. Selection of Microstructure Geometry

**Figure 3:**
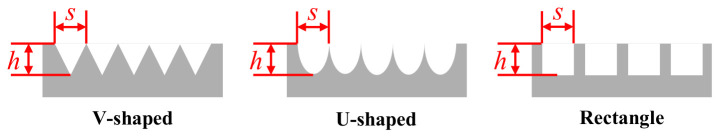
Cross-section shape of the groove.

This paper comprehensively considers the characteristics of laser processing and the existing striped grooves, and selects “V-shaped grooves”, “U-shaped grooves” and “rectangular grooves” to compare and analyze the drag-reduction effects of different types of grooves, as shown in Figure 3.

Different surface morphologies can be obtained by controlling the shape, depth and width-to-depth ratio of the grooves. The model is divided into three groups according to the groove shape. Each group is simulated by setting dimensionless depth and width-to-depth ratio as variables to explore the drag-reduction rate of different morphologies. The selected microstructure parameters are shown in Table 2.

#### 2.2.3. Identification of Drag-Reduction Performance

To study the drag-reduction effect of microstructures, it is necessary to construct a reasonable identification method and evaluation criterion to accurately determine their drag-reduction performance. In the plate flow-around model, the resistance on the plate surface includes pressure difference resistance and frictional resistance. When the surface shape changes, it has a significant impact on the pressure difference resistance. On the longitudinal microstructure surface, since the surface shape has not changed, differential pressure resistance is not the main source of resistance. In contrast, frictional resistance is the main factor influencing resistance. Therefore, in this paper, the drag-reduction rate η is determined by comparing the frictional resistance:

(13) $$\eta =\frac{F_{f0}-F_{f}}{F_{f0}}\times 100\%$$

In the formula, $F_{f0}$ represents the frictional resistance of the smooth plate surface, and $F_{f}$ represents the frictional resistance of the surface with microstructure. $F_{f}$ can be expressed as follows:

(14) $$F_{f}=C_{f}\frac{\rho u_{0}^{2}}{2}A_{f}$$

In the formula, $A_{f}$ is the surface area; $C_{f}$ is the coefficient of frictional resistance, which is expressed by the local coefficient of frictional resistance $C_{fx}$, that is, the ratio of the shear stress $\tau_{0}$ at position x on the surface to the kinetic energy of $0.5\rho u_{0}^{2}$ per unit volume of the incoming fluid:

(15) $$C_{fx}=\frac{\tau_{0}}{0.5\rho u_{0}^{2}}$$

Substituting Equation (5) into the above equation, we get:

(16) $$C_{fx}=\frac{0.074}{{Re}_{x}^{1/5}}$$

### 2.3. Simulation of Drag-Reduction Performance

#### 2.3.1. Selection of the Computational Domain Size

**Figure 4:**
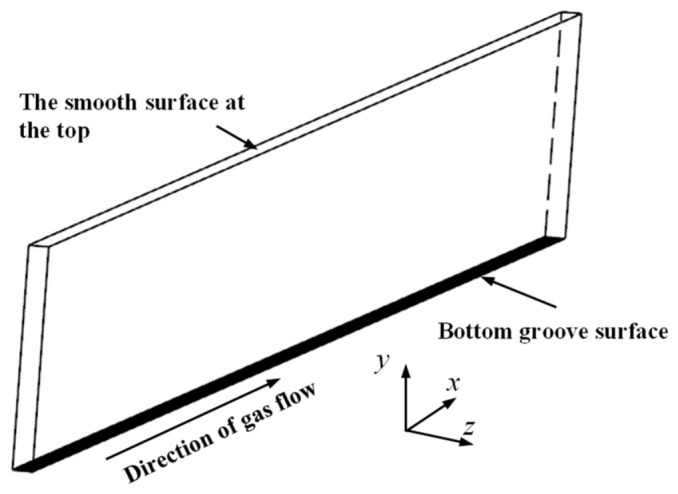
Schematic diagram of the computational domain model.

For the flat plate flow-around model, when the Reynolds number is greater than 1 × 106, the flow field within the boundary layer will develop into turbulence. In this paper, the air flow velocity is set at 100 m/s. According to the Reynolds number calculation formula of the plate flow-around the model, the model length at this time is at least 292 mm. To ensure the full development of turbulence in the calculation domain and reduce the calculation cost, the length *x* of the simulation calculation domain is set to 300 mm. From the boundary layer thickness Formula (6), it can be known that when *x* = 300 mm, the boundary layer thickness is the maximum, and at this time, the boundary layer thickness is 6.97 mm. To avoid mutual interference between the top and bottom boundary layers during the simulation process, the height *y* of the simulation calculation domain is set to 20 mm. The top of the computational domain is a smooth surface, and the bottom width *z* is uniformly set to the widths of 10 grooves, as shown in Figure 4.

#### 2.3.2. Grid Division of the Computational Domain Model

**Figure 5:**
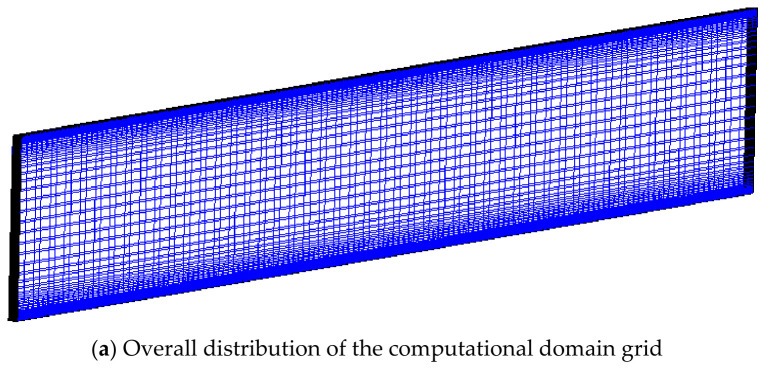

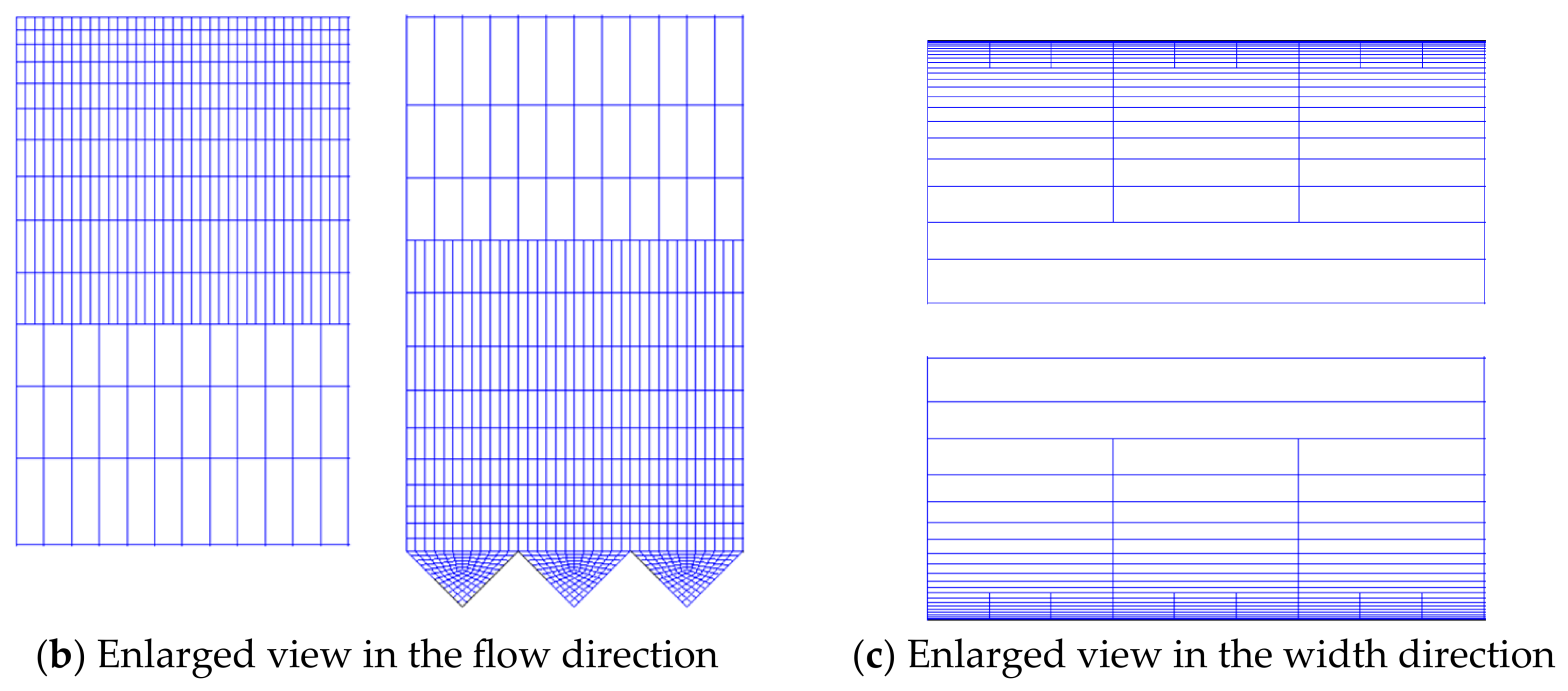
Computational domain grid.

The ICEM software (version 14.0) is adopted to conduct hierarchical grid division of the flow field area, which can reduce the computational load while ensuring the calculation accuracy. Grid encryption processing is carried out in the areas close to the top and bottom of the computational domain in the height direction of the computational domain. Among them, the V-shaped slots are meshed using the Y-block method. The following is shown in Figure 5: (a) the overall distribution of the computational domain grid; (b) the left side shows an enlarged view of the top smooth surface grid in the direction of flow, and the right side shows an enlarged view of the V-shaped groove grid at the bottom in the direction of flow; (c) enlarged view of the top smooth surface grid in the width direction on the upper side, and enlarged view of the V-shaped groove grid at the bottom in the width direction on the upper side.

#### 2.3.3. Turbulence Model and Boundary Conditions

At present, turbulence numerical simulation methods are divided into two types: Direct Numerical Simulation (DNS) and indirect numerical simulation. The commonly adopted non-direct numerical simulation methods include Large Eddy Simulation (LES) and Reynolds Averaged Navier-Stokes (RANS) [18].

However, the DNS and LES methods involve large computational loads and require extremely high processing power and memory space from the computer, making them difficult to be widely applied in practical engineering fields. The RANS method addresses the turbulent effects by decomposing the physical quantities (such as velocity, concentration, etc.) in the turbulence into average values and perturbation (fluctuation) components [19]. Specifically, the instantaneous quantities of turbulence are expressed as a time-averaged quantity and a deviation from the average value. Then, for these decomposed quantities, the governing equations are temporally averaged to obtain the equations describing the average flow. To further capture the influence of turbulence, turbulence models are usually introduced to simulate the additional effects of turbulence on the flow. Since this method only focuses on calculating the average flow state and omits the precise description of turbulence details, it results in a significant reduction in computational effort compared to directly simulating turbulence. Therefore, the Reynolds averaging method has become one of the most commonly used turbulence simulation methods at present.

In this paper, the turbulence simulation is carried out using the Realized k-ε model in the RANS model based on Reynolds averaging. This model can be accurately solved in the turbulent development region, but has poor adaptability to the region with a lower Reynolds number. Therefore, when using the Realized k-ε model, a wall function needs to be added for processing. Fluent software (version 2024 R1) offers three different wall functions, among which this paper selects the enhanced wall function. Although this function has high requirements for the quality of the grid, it takes into account the viscous influence of the fluid near the wall and is particularly suitable for the situation of low Reynolds number flow near the wall.

To compare the drag-reduction effects of microstructures with different structures and parameters, it is necessary to set unified boundary conditions, as shown in Table 3.

#### 2.3.4. Verification of Computational Model Accuracy

To verify the rationality of the model and mesh selection in this section, a smooth plate area was selected for simulation analysis. In the computational domain model, starting from the entrance, the simulated values of the frictional resistance coefficient are extracted every 50 mm for comparison and analysis with the theoretical values, totaling 5 points, to verify the accuracy of the model. The empirical formula for the frictional resistance coefficient of the flat plate model is given by Equation (16), and Table 4 lists the theoretical and simulated values of the frictional resistance coefficient at different positions.

**Figure 6:**
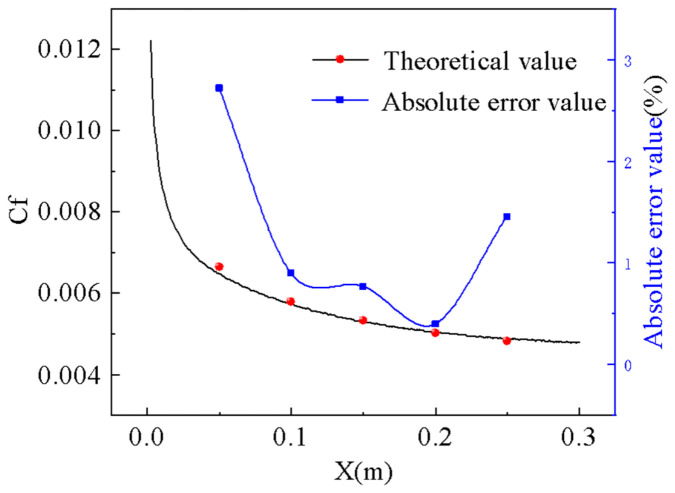
Comparison between the simulated value and the theoretical value of the coefficient of friction resistance.

As shown in Figure 6, the difference between the simulated value and the theoretical value of the friction resistance coefficient is compared. It can be seen from the figure that the simulated value and the theoretical value are highly coincident, with the maximum error being 2.72%. Furthermore, by analyzing the images, it can be seen that the errors at the inlet and outlet of the computational domain model are relatively large. This is because the fluid at the inlet is in a laminar flow state and cannot be accurately calculated using the empirical formula for the turbulent friction resistance coefficient. At the outlet, the flow conditions are affected by the outlet; therefore, the errors at both locations are relatively large, but overall, they are acceptable. Therefore, it can be indicated that this method can meet the requirements of calculation accuracy and can be used to study the drag-reduction effect on the groove surface.

The smooth plate validation confirms the reliability of the flow field framework and boundary layer development. The secondary vortex structures and low-velocity regions inside grooves obtained in this study are consistent with the typical flow characteristics of riblet surfaces reported in published literature, which further supports the credibility of the simulation in capturing complex near-wall physics.

### 2.4. Analysis of Simulation Results

To systematically evaluate the drag-reduction characteristics of different groove morphologies, numerical simulations were conducted for V-shaped, U-shaped, and rectangular groove microstructures under various geometric parameter combinations. Based on the drag-reduction rate calculated by Equation (13), the effects of groove depth and aspect ratio on aerodynamic performance were quantitatively analyzed. Furthermore, the drag-reduction mechanisms of different groove geometries were clarified by combining wall shear stress distributions, streamwise velocity contours, and spanwise vortex structures. Through comparative analysis of the three groove morphologies, the optimal groove geometry and its corresponding hydrodynamic regulation mechanism were identified.

The simulation results indicate that although all three groove structures exhibit measurable drag-reduction capability, their performances differ significantly due to variations in geometric morphology. This difference is mainly reflected in their abilities to trap near-wall low-momentum fluid, regulate local velocity gradients, and induce stable secondary vortical structures. These coupled flow-control effects ultimately determine the drag-reduction efficiency of the structured surface.

#### 2.4.1. Analysis of V-Shaped Groove Simulation Results

**Figure 7:**
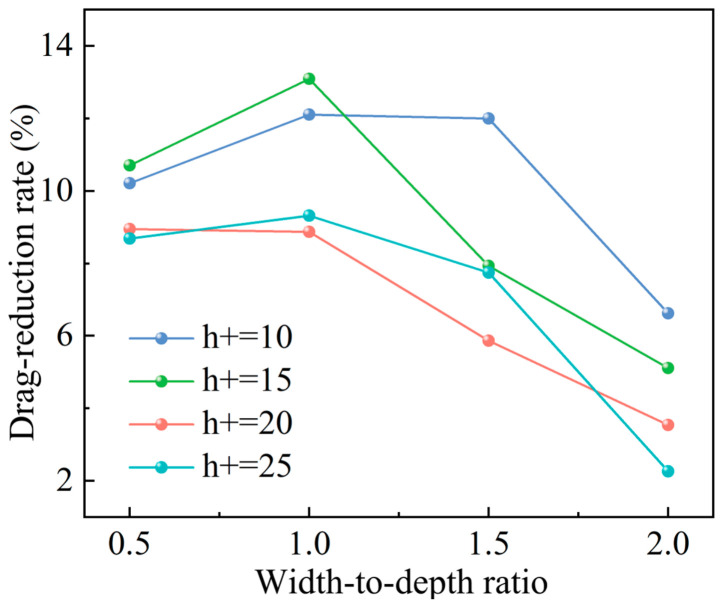
The influence law of V-shaped groove structure parameters on the drag-reduction rate.

The drag-reduction performance of V-shaped grooves under different geometric parameters is summarized in Table 5, and the corresponding variation trends are shown in Figure 7. It can be observed that the drag-reduction rate exhibits a distinct trend of first increasing and then decreasing with increasing aspect ratio (AR) under the same groove depth condition. Among all investigated cases, the maximum drag-reduction rate reaches 13.1% when the dimensionless groove depth is *h^+^* = 15 and the aspect ratio is 1.0, indicating that this geometric configuration provides the optimal flow-regulation effect.

As shown in Figure 7, when the aspect ratio increases from 0.5 to 1.0, the drag-reduction rate increases significantly. This indicates that moderate widening of the groove cavity enhances the groove’s capability to accommodate and retain low-momentum fluid, thereby improving hydrodynamic isolation between the wall surface and the high-speed mainstream flow. However, when the aspect ratio continues to increase beyond 1.0, the drag-reduction rate gradually decreases. This suggests that excessive widening weakens the sidewall confinement effect, allowing for stronger turbulent penetration into the groove cavity and increasing local momentum exchange near the wall. Therefore, the drag-reduction effect of V-shaped grooves is not simply proportional to groove width, but depends on an optimal geometric matching relationship between groove depth and groove opening width.

**Figure 8:**
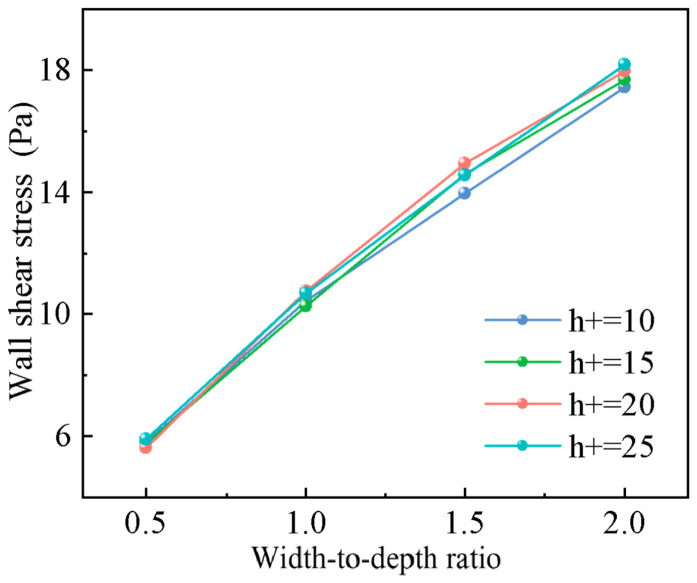
Influence law of V-shaped groove structure parameters on wall shear stress.

The variation of wall shear stress is further illustrated in Figure 8. It can be seen that under the optimal aspect ratio condition, the wall shear stress reaches a relatively low level compared with other configurations. This indicates that the groove geometry under this condition most effectively reduces near-wall viscous friction. Moreover, although some geometric conditions also exhibit reduced wall shear stress, their drag-reduction performance is not equally significant, implying that drag reduction is governed not only by local wall shear stress reduction, but also by the overall regulation of the near-wall flow field.

**Figure 9:**
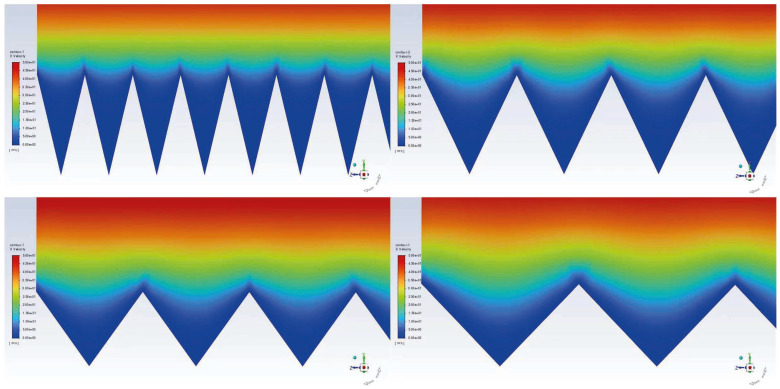
Surface velocity cloud diagrams of V-shaped grooves with different width-to-depth ratios.

To further clarify this mechanism, the streamwise velocity contours in Figure 9 were analyzed. A distinct low-velocity region can be observed inside the V-shaped groove cavity, and this low-speed region becomes more pronounced under the optimal configuration of *h^+^* = 15 and AR = 1.0. The enlarged low-speed zone indicates that the groove has a stronger ability to trap low-momentum fluid and form a relatively stable fluid buffer layer near the wall. This fluid layer effectively separates the wall from the high-speed outer flow, thereby reducing the wall-normal velocity gradient and weakening viscous momentum transfer. As a result, wall friction is significantly reduced, leading to improved drag-reduction performance.

**Figure 10:**
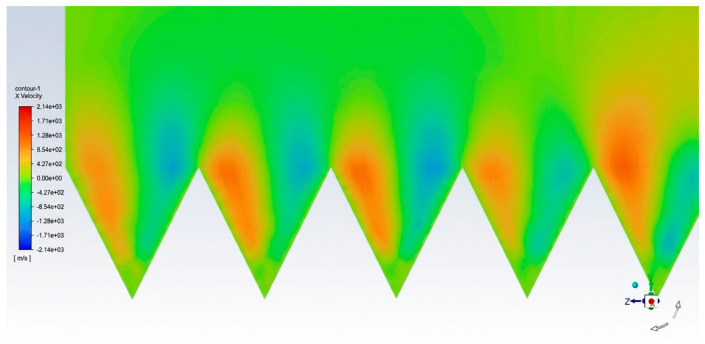
Z—direction velocity cloud diagram of the V—shaped groove surface.

In addition, the spanwise velocity distributions shown in Figure 10 reveal the formation of relatively organized secondary vortical structures above the groove crest region. Under the optimal geometric condition, the counter-rotating vortices are more concentrated and symmetric, indicating better vortex stability and stronger spanwise momentum redistribution capability. These organized vortices not only help maintain the stability of the low-speed buffer layer inside the groove cavity, but also suppress turbulent bursting events near the wall and weaken momentum transport from the outer turbulent region toward the viscous sublayer. Consequently, both viscous drag and turbulence-induced frictional dissipation are reduced simultaneously.

Therefore, based on the combined evidence from Figure 7, Figure 8, Figure 9 and Figure 10, the superior drag-reduction performance of the V-shaped groove can be attributed to three synergistic effects: enhanced low-speed fluid entrapment, effective reduction in wall shear stress, and stable secondary vortex generation for turbulence suppression. This coupling mechanism enables the V-shaped groove to exhibit the strongest hydrodynamic regulation capability among the investigated groove geometries.

#### 2.4.2. Analysis of U-Shaped Groove Simulation Results

**Figure 11:**
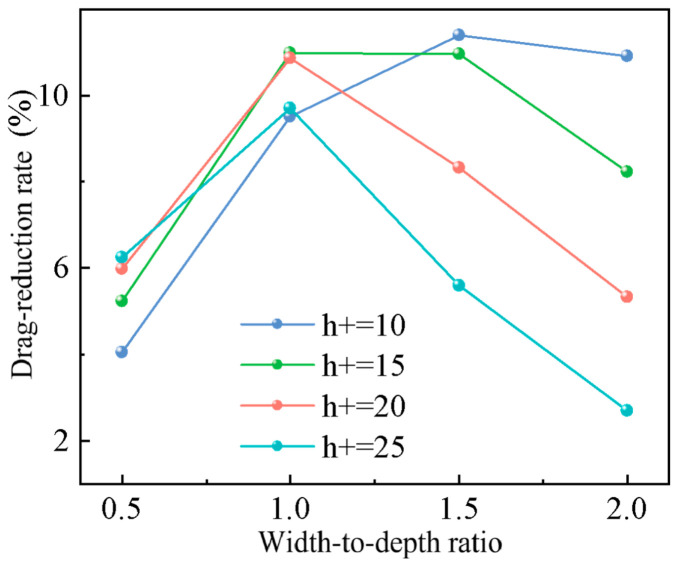
The influence law of U-shaped groove structure parameters on the drag-reduction rate.

The simulation results of the U-shaped groove are summarized in Table 6, and the corresponding drag-reduction variation is shown in Figure 11. Similar to the V-shaped groove, the drag-reduction rate of the U-shaped groove also exhibits a trend of first increasing and then decreasing with increasing aspect ratio under the same dimensionless depth. The maximum drag-reduction rate reaches 11.39% at *h^+^* = 15 and an aspect ratio of 1.0, which indicates that the U-shaped groove also possesses an optimal geometric matching relationship between groove depth and width. However, its maximum drag-reduction performance is still lower than that of the V-shaped groove.

As shown in Figure 11, when the aspect ratio increases from 0.5 to 1.0, the drag-reduction rate increases noticeably, indicating that a moderate increase in groove width improves the groove cavity’s ability to retain low-momentum fluid and regulate the near-wall flow field. This behavior is consistent with the V-shaped groove. Nevertheless, when the aspect ratio further increases, the drag-reduction rate gradually declines, suggesting that an excessively large groove opening weakens the confinement effect of the groove cavity and allows for stronger mainstream intrusion into the groove region, thereby increasing local momentum exchange and reducing drag-reduction efficiency.

**Figure 12:**
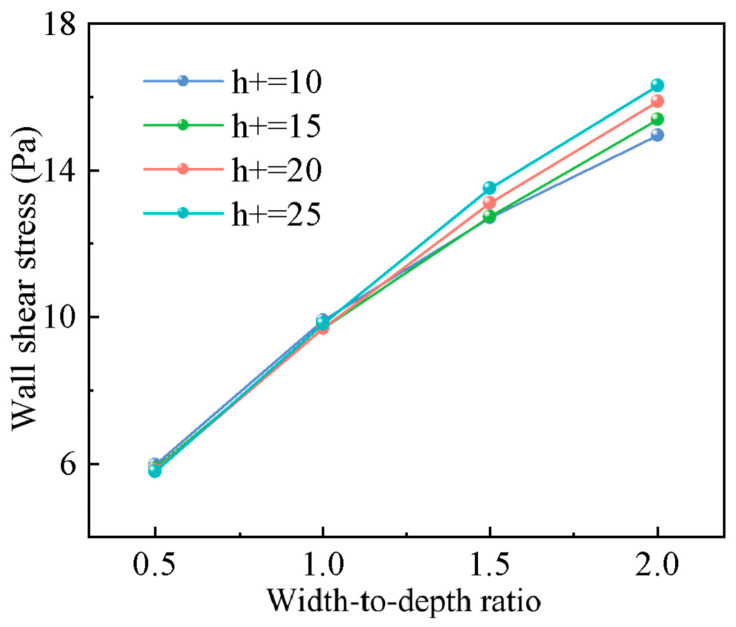
The influence law of U-shaped groove structure parameters on wall shear stress.

The wall shear stress distribution presented in Figure 12 further supports this trend. Under the optimal parameter condition, the wall shear stress reaches a relatively low value, confirming that the U-shaped groove can effectively reduce near-wall viscous dissipation. However, compared with the corresponding V-shaped configuration, the reduction amplitude is smaller, indicating that the U-shaped groove has a relatively weaker ability to regulate local shear stress distribution. This difference suggests that groove profile geometry plays an important role in determining the intensity of wall–flow interaction.

**Figure 13:**
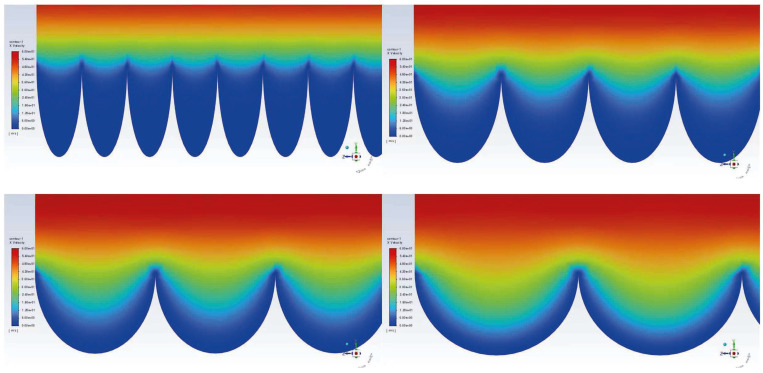
Surface velocity cloud diagrams of U-shaped grooves with different width-to-depth ratios.

The streamwise velocity contours shown in Figure 13 indicate that a low-velocity region is also formed inside the U-shaped groove cavity. Compared with the V-shaped groove, the low-speed region inside the U-shaped groove is relatively broader but less concentrated. This implies that although the curved groove profile can retain low-momentum fluid, its confinement capability is weaker, making the trapped fluid layer less stable under the disturbance of the mainstream flow. Consequently, the fluid-buffering effect between the wall and outer flow is reduced, leading to a less significant decrease in the wall-normal velocity gradient.

**Figure 14:**
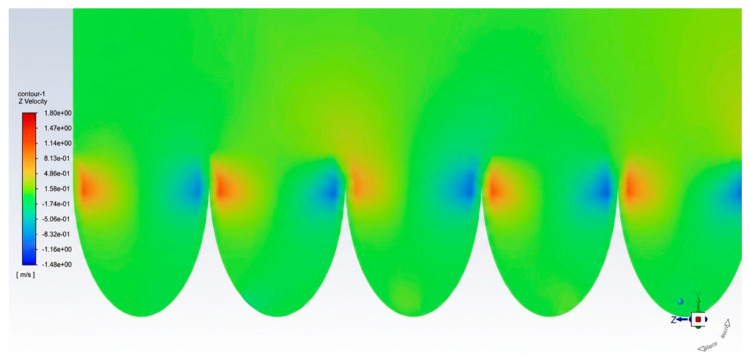
Z—direction velocity cloud diagram of the U—shaped groove surface.

Further analysis of the spanwise velocity distribution in Figure 14 shows that secondary vortical structures are also generated above the groove crest. However, compared with the V-shaped groove, the vortex core in the U-shaped groove is more dispersed and the rotational structure is less concentrated, indicating relatively weaker vortex stability. This suggests that the U-shaped groove has a lower ability to redistribute spanwise momentum and suppress turbulent bursting events near the wall. As a result, its turbulence suppression effect is weaker than that of the V-shaped groove.

Therefore, based on the combined analysis of Figure 11, Figure 12, Figure 13 and Figure 14, the drag-reduction mechanism of the U-shaped groove is mainly attributed to the formation of a near-wall low-speed buffer region and the induced secondary vortex motion. However, due to its relatively weak flow-convergence capability and lower vortex stability, its overall hydrodynamic regulation effect is weaker than that of the V-shaped groove, resulting in lower drag-reduction performance.

#### 2.4.3. Analysis of Simulation Results of Rectangular Grooves

**Figure 15:**
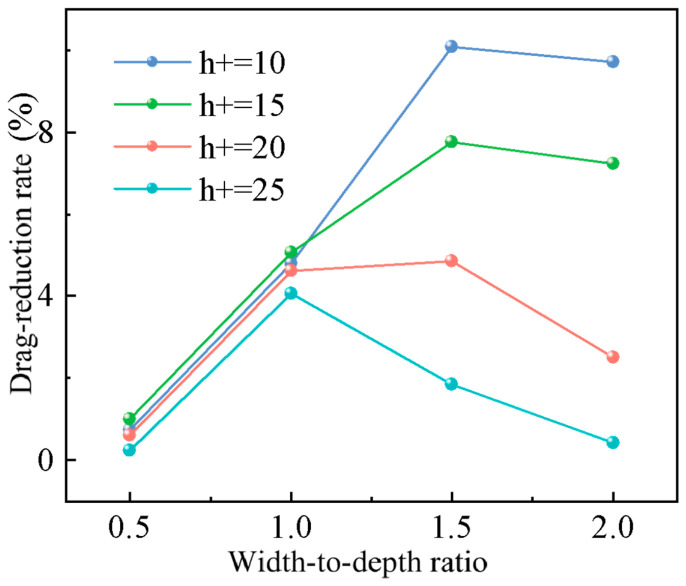
The influence law of rectangular groove structure parameters on drag reduction rate.

The drag-reduction performance of rectangular grooves under different geometric parameters is listed in Table 7, and the variation trend is shown in Figure 15. Similar to the previous two groove morphologies, the drag-reduction rate of the rectangular groove also shows a trend of first increasing and then decreasing with increasing aspect ratio. The maximum drag-reduction rate reaches 10.08%, which is lower than that of both V-shaped and U-shaped grooves, indicating that the rectangular groove exhibits the weakest drag-reduction capability among the three investigated structures.

As shown in Figure 15, when the aspect ratio increases to an appropriate range, the drag-reduction rate improves due to enhanced fluid accommodation inside the groove cavity. However, the increase is relatively limited, and the subsequent decline becomes more obvious when the aspect ratio continues to increase. This indicates that although the rectangular groove can provide internal space for low-momentum fluid retention, its geometric profile is less favorable for sustained flow regulation compared with the inclined or curved groove geometries.

**Figure 16:**
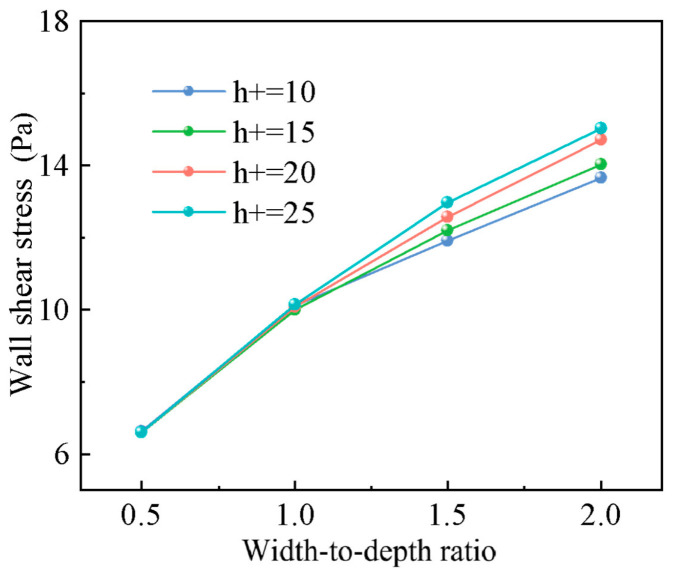
The influence law of rectangular groove structure parameters on wall shear stress.

The wall shear stress distribution shown in Figure 16 indicates that the rectangular groove can reduce wall shear stress under certain parameter combinations, but the reduction degree is relatively small compared with the V-shaped and U-shaped grooves. This suggests that the rectangular geometry has a weaker ability to reduce local viscous momentum transfer near the wall.

**Figure 17:**
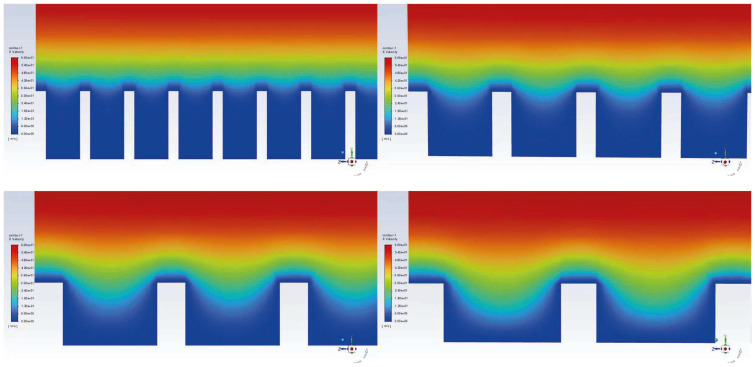
Surface velocity cloud map of rectangular grooves with different width-to-depth ratios.

The streamwise velocity contours in Figure 17 reveal that a low-velocity recirculation region is formed inside the rectangular groove cavity; however, the low-speed region is mainly concentrated near the bottom corners of the groove and is distributed unevenly. Compared with the more continuous low-speed fluid layer observed in V-shaped grooves, the trapped low-momentum fluid in rectangular grooves is less stable and provides weaker flow isolation between the wall and the outer mainstream flow. As a result, the reduction in wall-normal velocity gradient is limited.

**Figure 18:**
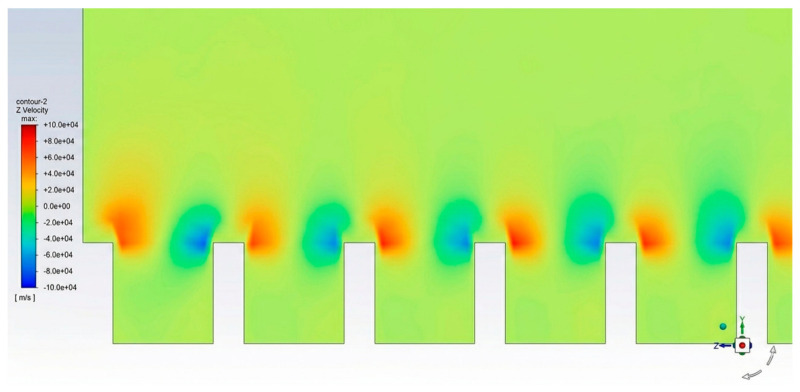
Z—direction velocity cloud map of the surface of the rectangular groove.

The spanwise velocity distributions shown in Figure 18 further demonstrate that vortical structures are generated above the rectangular groove. However, due to the sharp right-angle corners, local flow separation near the groove edges becomes more pronounced, causing the vortex structures to be more irregular and dispersed. Unlike the relatively organized counter-rotating vortices observed in V-shaped grooves, the vortical motion in rectangular grooves exhibits weaker coherence and lower stability, making it less effective in redistributing spanwise momentum and suppressing turbulent bursting near the wall.

Therefore, based on the combined analysis of Figure 15, Figure 16, Figure 17 and Figure 18, the relatively poor drag-reduction performance of rectangular grooves is mainly caused by weaker low-speed fluid confinement, limited wall shear stress reduction, and unstable secondary vortex structures induced by sharp-corner flow separation. These factors jointly lead to the lowest hydrodynamic regulation capability among the three groove geometries.

#### 2.4.4. Comparative Discussion and Optimal Structure Selection

A comprehensive comparison of the simulation results shows that the drag-reduction performance follows the order: V-shaped groove > U-shaped groove > Rectangular groove.

This difference is clearly supported by the combined evidence from the drag-reduction rate, wall shear stress, streamwise velocity field, and spanwise vortex structure analyses. The V-shaped groove exhibits the highest drag-reduction rate (13.1%), the most pronounced low-speed fluid region, the lowest effective wall shear stress level, and the most stable counter-rotating vortex structures. These characteristics indicate that the converging sidewall geometry of the V-shaped groove provides stronger hydrodynamic confinement and more effective turbulence regulation.

In comparison, although the U-shaped groove can also generate low-speed fluid regions and secondary vortices, its weaker flow-convergence capability results in lower vortex stability and weaker suppression of near-wall turbulent momentum transport. The rectangular groove further suffers from sharp-corner flow separation, unstable vortex structures, and limited low-speed fluid confinement, leading to the weakest drag-reduction performance.

Therefore, considering both drag-reduction efficiency and flow-regulation mechanism, the V-shaped groove with *h^+^* = 15 and AR = 1.0 is identified as the optimal microstructure geometry. This structure provides the most favorable coupling between low-speed fluid entrapment, wall shear stress reduction, and organized secondary vortex generation, making it the preferred configuration for subsequent femtosecond laser fabrication and experimental validation.

## 3. Results and Discussion

Based on the work presented in the previous section, this section first investigates the effects of processing parameters, including laser power, number of scans, and processing surface width, on the geometry of drag-reduction microstructures through single-factor experiments, and determines the feasible parameter range for fabricating the target microstructure. Subsequently, a regression prediction model is established using the Response Surface Methodology (RSM). After verifying the accuracy of the model, the processing parameters are optimized to satisfy the fabrication requirements of the target drag-reduction microstructure. Finally, verification experiments are carried out, and the morphology and surface quality of the optimized drag-reduction microstructure are systematically analyzed.

### 3.1. Experimental Equipment

The femtosecond laser used in the experiment (Pharos 6-600-PP) was manufactured by Light Conversion Company from Vilnius, Lithuania. This type of femtosecond laser can generate lasers at three wavelengths: 1026 nm, 513 nm, and 342 nm. During the experiment, the laser wavelength used was 1026 nm, the pulse width of the laser was 200 fs, the repetition frequency was 200 kHz, and the diameter of the focused light spot was approximately 30 μm.

During the experiment, in order to measure the surface topography and structural dimensions after femtosecond laser processing, the InfiniteFocus automatic zoom three-dimensional surface measurement instrument from the Austrian company Bruker Alicona in Graz was used. This instrument utilizes the optical measurement principle of a high-power microscope to adjust the distance between the object and the microscope in real time. It conducts layer-by-layer scanning at different focusing points and combines a precise positioning system for data collection. By obtaining multiple measurement points at different heights, the instrument can reconstruct the three-dimensional surface topography of the sample, thereby achieving precise surface measurement and analysis.

### 3.2. Selection of Process Parameter Range

Based on previous experimental experience in femtosecond laser scribing of Ti6Al4V, the groove width generated by a single scanning path is generally less than 30 μm, while the groove depth is typically less than 10 μm. Therefore, to fabricate the drag-reduction microstructure designed in Section 2, a multiple parallel scribing strategy was adopted to satisfy the dimensional requirements of the target groove. In this study, the path width of femtosecond laser processing is defined as the face width (S). The groove width is mainly related to the face width, whereas the groove depth is primarily affected by laser power and the number of repeated scans. The scanning speed was fixed at 500 mm/s. Single-factor experiments were conducted to determine the reasonable range of processing parameters, and the experimental scheme is listed in Table 8.

**Figure 19:**
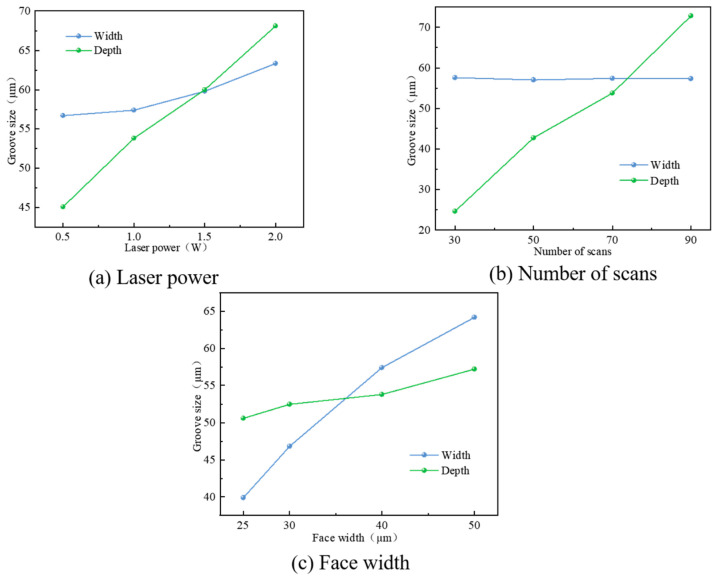
The influence of a single factor on the size of the drag-reduction groove.

Figure 19 illustrates the influence of single processing factors on groove geometry. As shown in Figure 19a, both groove width and groove depth increase with increasing laser power. This is mainly because higher laser power increases the pulse energy delivered to the Ti6Al4V surface, thereby enhancing laser-material interaction intensity and material removal efficiency. Consequently, both the lateral removal range and vertical ablation depth increase simultaneously. Considering that the target groove depth of 54 μm should be located within the middle range of the process window to facilitate subsequent optimization, laser powers of 0.5 W, 1.0 W, and 1.5 W were selected for response surface experiments.

As shown in Figure 19b, the groove width remains nearly unchanged with increasing number of scans, whereas groove depth increases significantly. This indicates that repeated scanning mainly contributes to cumulative material removal in the depth direction, while its influence on lateral groove expansion is relatively limited under a fixed face width condition. Based on the dimensional requirement of the target groove depth, 50, 70, and 90 scans were selected for the response surface design.

As shown in Figure 19c, groove width increases significantly with increasing face width, while groove depth changes only slightly. This is because face width directly determines the lateral processing path of femtosecond laser scanning and therefore has a dominant influence on groove opening width, whereas its effect on vertical energy deposition is comparatively weaker. To ensure that the target groove width approaches 54 μm while maintaining the selected parameter level within the middle range of the process window, face widths of 30 μm, 40 μm, and 50 μm were chosen for the subsequent response surface experiments.

Overall, the single-factor results indicate that laser power simultaneously affects groove width and groove depth, the number of scans mainly dominates groove depth, and face width primarily determines groove width, which provides an important basis for subsequent multi-factor optimization.

### 3.3. Drag-Reduction Microstructure Processing

#### 3.3.1. Experimental Scheme of Response Surface Method

The Response Surface Methodology (RSM) is a statistical and mathematical optimization technique used to establish predictive models for exploring the relationship between multiple independent variables and response variables. Owing to its high efficiency in process optimization, RSM was employed in this study to analyze the interactive effects of process parameters on drag-reduction groove geometry and to optimize the femtosecond laser processing parameters for target microstructure fabrication. The factor-level design of the response surface experiments is shown in Table 9.

#### 3.3.2. Regression Prediction Model

The experimental results were analyzed using Design-Expert software (version 13), and quadratic regression models for groove width (W) and groove depth (H) were established through multiple regression analysis, as expressed in Equations (17) and (18). Analysis of variance (ANOVA) was subsequently performed for both models, and the results are presented in Table 10 and Table 11.

(17) $$\begin{array}{c} W=35.22375+1.94P-0.07075n+0.0665S-0.0125Pn-0.04PS+0.000125nS+1.68P^{2}\\ +0.0007375n^{2}+0.0107S^{2}\; \end{array}$$

(18) $$\begin{array}{c} H=56.57125+13.545P-0.609125n-1.61425S+0.275Pn+0.015PS+0.002375Ns-8.96P^{2}\\ +0.007525n^{2}+0.02185S^{2} \end{array}$$

As shown in Table 10 and Table 11, both regression models are statistically significant (*p* < 0.0001), indicating that the established models can effectively describe the relationship between processing parameters and groove geometry. In addition, the lack-of-fit terms are not significant, which confirms that both models exhibit good adequacy within the selected experimental range.

The high correlation coefficients further demonstrate the reliability of the developed models. For groove width, the model exhibits R^2^ = 0.9965, *R*^2^*adj* = 0.9920, and *R*^2^*pre* = 0.9867, with a coefficient of variation of only 1.03%. For groove depth, the corresponding values are 0.9777, 0.9491, and 0.8830, respectively, with a coefficient of variation of 5.62%. The high fitting coefficients, together with the small differences between adjusted and predicted coefficients, indicate that both models possess good fitting quality and reliable predictive capability.

More importantly, the ANOVA results reveal clear differences in factor significance. For groove width, the significance order of the processing parameters is: Face width > Laser power > Number of scans. Among them, face width has the most significant influence, which is consistent with the physical processing mechanism, since face width directly determines the lateral scanning path and therefore dominates groove opening width. Laser power has a secondary influence by affecting the effective ablation range, whereas repeated scanning has only a limited effect on lateral dimensional expansion.

For groove depth, the significance order is as follows: Number of scans > Laser power > Face width. This indicates that vertical groove formation is mainly controlled by cumulative material removal during repeated scanning. Laser power further affects the ablation efficiency of each scan, while face width mainly changes lateral energy distribution and has a relatively weak influence on vertical energy deposition.

These results quantitatively confirm the physical trends observed in the single-factor experiments and provide a reliable theoretical basis for subsequent parameter optimization.

#### 3.3.3. Verification of Regression Prediction Models

**Figure 20:**
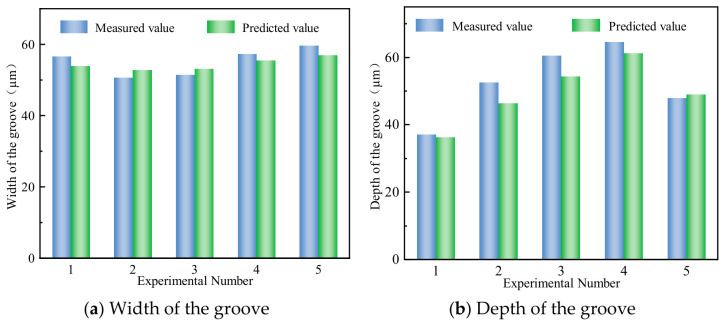
Comparison of regression prediction results.

To verify the predictive accuracy of the regression models, five groups of experiments were selected within the prediction interval for validation, and the experimental scheme is listed in Table 12. The comparison between predicted values and experimental results is shown in Figure 20.

As shown in Figure 20, the predicted results are in good agreement with the experimental measurements for both groove width and groove depth. The maximum prediction errors are 4.6% and 11.8%, respectively, while the average errors of the five experimental groups are only 2.74% for groove width and 6.32% for groove depth.

These relatively small deviations indicate that the developed regression models possess good prediction accuracy and engineering reliability. Therefore, the established models can provide effective parameter guidance for the precise fabrication of the target drag-reduction microstructure, thereby improving process optimization efficiency and reducing trial-and-error costs in practical fabrication.

#### 3.3.4. Response Surface Analysis

**Figure 21:**
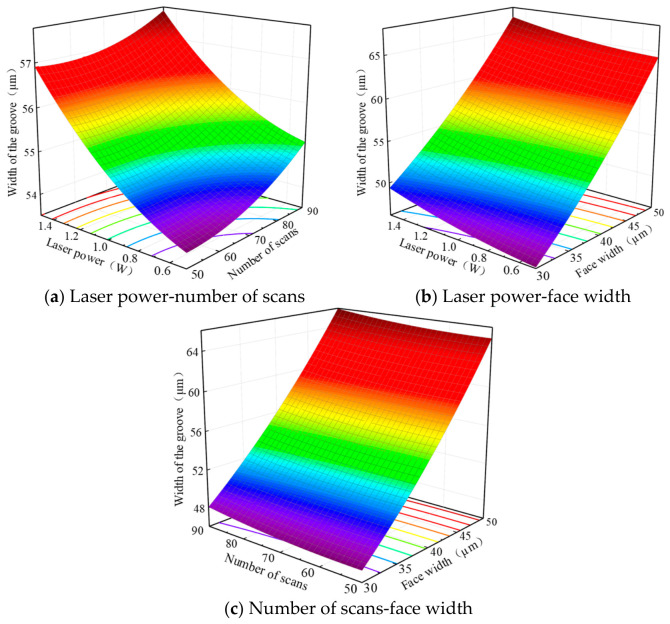
Analysis of the response surface of groove width.

**Figure 22:**
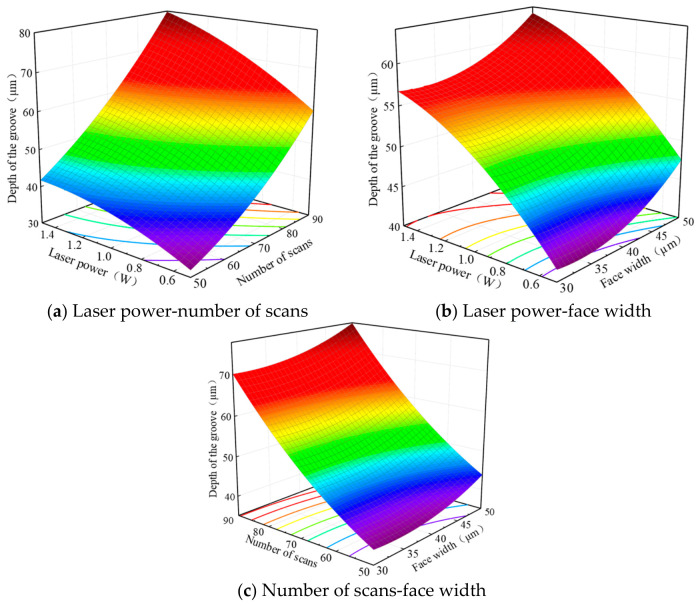
Analysis of the groove depth response surface.

According to the regression prediction models, groove width and groove depth are interactively influenced by laser power, number of repeated scans, and face width. To more intuitively illustrate the effects of these processing parameters on groove geometry, response surface plots were generated by fixing one factor and analyzing the interactive influence of the other two factors, as shown in Figure 21 and Figure 22.

Figure 21 presents the response surface analysis of groove width. As shown in Figure 21a, groove width increases with increasing laser power and number of scans. However, when laser power is kept constant, the influence of repeated scanning on groove width is relatively weak. This is because under a fixed face width condition, the lateral processing path remains unchanged, and repeated scanning mainly contributes to cumulative vertical material removal rather than lateral expansion. In contrast, when the number of scans is constant, groove width increases more noticeably with increasing laser power, indicating that higher pulse energy enlarges the effective lateral ablation range and enhances groove widening. This further confirms that the influence of laser power on groove width is greater than that of repeated scanning.

As shown in Figure 21b, groove width increases significantly with increasing laser power and face width, while the influence of face width is particularly prominent. This is because face width directly determines the lateral scanning path width during femtosecond laser processing, thereby exerting a dominant influence on the final groove opening width. Laser power acts mainly by modifying the ablation intensity and lateral thermal-affected removal range, but its contribution is still smaller than that of face width.

Figure 21c further shows that the influence of repeated scanning on groove width is much smaller than that of face width. Compared with the relatively weak contribution of repeated scanning, increasing face width produces a much more significant widening effect. By comparing the three response surfaces, it can be concluded that the significance order of processing parameters affecting groove width is as follows: Face width > Laser power > Number of scans.

This result is in good agreement with the ANOVA analysis presented in Table 10. When the laser power is around 1.0 W, the number of scans is approximately 80, and the face width is around 40 μm, the processing requirement of a V-shaped groove width of 54 μm can be satisfied.

Figure 22 shows the response surface analysis of groove depth. As shown in Figure 22a, groove depth gradually increases with increasing laser power and repeated scanning times. Compared with laser power, the influence of repeated scanning is more significant. This is mainly because repeated scanning promotes cumulative layer-by-layer material removal, resulting in continuous deepening of the groove. Although increasing laser power enhances single-pass ablation efficiency, its contribution to total vertical removal is still less significant than that produced by multiple scanning cycles.

As shown in Figure 22b, groove depth increases with increasing laser power and face width, but the influence of face width is relatively limited compared with laser power. This is because face width mainly changes the lateral distribution of laser energy, whereas vertical ablation depth is primarily determined by energy deposition in the depth direction. Therefore, increasing laser power more effectively enhances vertical material removal efficiency than changing face width.

Figure 22c further demonstrates that the influence of repeated scanning on groove depth is significantly greater than that of face width. The groove depth increases markedly with increasing scanning times, whereas changes in face width produce only a relatively weak effect. By comparing the three response surfaces, it can be concluded that the significance order of processing parameters affecting groove depth is as follows: Number of scans > Laser power > Face width.

This conclusion is consistent with the regression analysis results shown in Table 11. When the laser power is around 0.9 W, the number of scans is approximately 70, and the face width is around 40 μm, the processing requirement of a V-shaped groove depth of 54 μm can be satisfied.

#### 3.3.5. Optimization of Process Parameters

Design-Expert software was further employed to optimize the processing parameters. The minimum groove width, maximum groove width, minimum groove depth, and maximum groove depth were taken as optimization objectives, respectively. The optimization results are listed in Table 13.

As shown in Table 13, within the effective prediction range of the developed regression models, the achievable groove width ranges from 34.2 μm to 66.7 μm, while the groove depth ranges from 31.0 μm to 82.4 μm, indicating that the femtosecond laser processing window established in this study is sufficiently broad to cover the dimensional requirements of the target drag-reduction microstructure.

Based on the simulation results in Section 2, the target groove geometry was set to 54 μm in width and 54 μm in depth, corresponding to the optimal V-shaped drag-reduction microstructure. By simultaneously constraining groove width and groove depth to the target values, the optimized processing parameter combination was determined as follows: Laser power = 0.77 W, Number of scans = 77, Face width = 39 μm.

**Figure 23:**
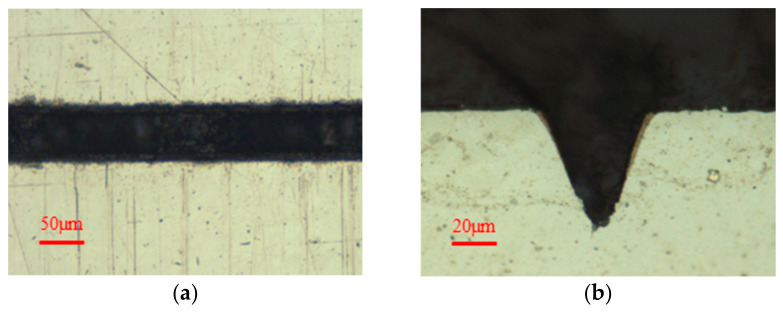
Surface morphology after optimization. (**a**) Surface morphology of the groove; (**b**) Cross-section morphology of the groove.

Figure 23 presents the fabricated groove morphology under the optimized processing parameters. It can be seen that the groove morphology is uniform and well defined, with an experimentally measured groove width of 55.9 μm and groove depth of 55.5 μm, which are in good agreement with the target dimensions. This indicates that the optimized parameter combination exhibits excellent dimensional controllability and can effectively satisfy the design requirement of a width-to-depth ratio of 1:1 proposed in Section 2.

As shown in Figure 23a, no obvious heat-affected zone is observed at the groove edge, indicating that femtosecond laser processing effectively suppresses thermal diffusion and minimizes thermal damage to the surrounding material due to its ultrashort pulse duration and highly localized energy deposition. As shown in Figure 23b, the cross-sectional morphology of the processed groove exhibits an approximately V-shaped contour, and the contour consistency is high along the processed profile, indicating excellent shape fidelity of the fabricated drag-reduction structure.

These results demonstrate that the optimized femtosecond laser processing parameters can not only accurately realize the target V-shaped groove geometry, but also effectively control thermal effects and maintain high structural consistency, thereby providing a reliable manufacturing basis for subsequent drag-reduction array fabrication.

### 3.4. Processing and Analysis of Array Drag-Reduction Microstructures

**Figure 24:**
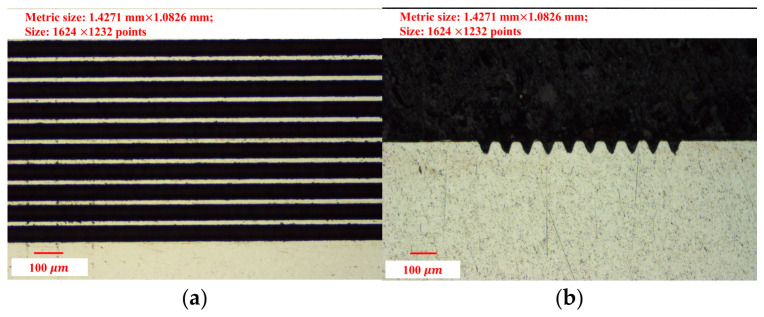
Surface morphology of the array drag-reduction structure. (**a**) Surface morphology of array grooves; (**b**) Cross-sectional morphology of the array groove.

Based on the optimized processing parameters obtained in Section 3.2, ten array drag-reduction structures were fabricated on the Ti6Al4V surface, as shown in Figure 24.

As shown in Figure 24, both the groove width and groove depth remain stable at approximately 55 μm, which is highly consistent with the designed target dimensions. Moreover, the groove surfaces and sidewalls are smooth and uniform, and the array grooves exhibit high geometric consistency over the processed area. This indicates that the optimized femtosecond laser processing parameters possess excellent process stability and repeatability, enabling high-quality batch fabrication of drag-reduction microstructures.

**Figure 25:**
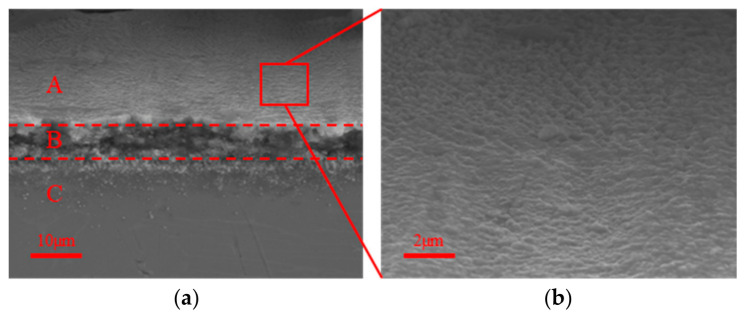
SEM image of the inner wall of the groove. (**a**) Surface topography of the groove edge; (**b**) Local enlargement of the inner wall of the groove.

To further evaluate the quality of the groove inner wall, scanning electron microscopy (SEM) characterization was conducted, and the results are shown in Figure 25. As shown in Figure 25a, region A corresponds to the inner wall of the groove, region B is the processed groove edge, and region C is the unprocessed Ti6Al4V substrate. Although a small amount of molten debris is observed near the processed edge, the inner wall of the groove remains relatively smooth and flat, indicating that the material removal process is highly localized and that excessive thermal accumulation is effectively suppressed during femtosecond laser ablation.

Figure 25b presents a locally enlarged view of the groove inner wall. It can be observed that the inner wall surface exhibits a relatively uniform granular microstructure, with homogeneous surface roughness and no obvious cracks, pores, or severe recast defects. This indicates that the optimized femtosecond laser processing parameters not only ensure dimensional accuracy and contour consistency, but also produce high-quality machined surfaces with minimal structural defects.

Overall, the results demonstrate that femtosecond laser processing, under optimized parameters, can achieve high-consistency, high-precision, and high-quality fabrication of array drag-reduction microstructures, providing strong technological support for the practical application of drag-reduction functional surfaces on Ti6Al4V components.

## 4. Conclusions

In this study, a systematic investigation on the design, simulation, and precision fabrication of drag-reduction microstructures on Ti6Al4V surfaces was conducted by integrating Computational Fluid Dynamics (CFD) analysis with femtosecond laser precision processing optimized via Response Surface Methodology (RSM). The main conclusions are summarized as follows:

(1) Among the three biomimetic groove geometries designed based on the boundary layer theory, namely the V-shaped groove, U-shaped groove, and rectangular groove, the V-shaped groove exhibited the best drag-reduction performance. When the dimensionless groove depth was *h*^+^ = 15 and the aspect ratio was 1:1, the maximum drag-reduction rate reached 13.1%. The superior drag-reduction performance is mainly attributed to the formation of a stable low-velocity fluid region inside the groove, which effectively thickens the viscous sublayer and suppresses near-wall turbulent bursting behavior through the action of secondary vortical structures, thereby reducing wall shear stress.

(2) Single-factor experiments and response surface analysis revealed the influence mechanisms of femtosecond laser processing parameters on groove geometry. The results indicate that laser power simultaneously affects groove width and groove depth, the number of scans mainly determines groove depth through cumulative vertical material removal, and face width primarily controls groove width by defining the lateral processing path. The developed quadratic regression models exhibited high fitting accuracy, with correlation coefficients (R^2^) of 0.9965 for groove width and 0.9777 for groove depth, respectively, demonstrating excellent predictive capability.

(3) For the target drag-reduction microstructure with a groove width of 54 μm and a groove depth of 54 μm, the optimal processing parameter combination was determined to be 0.77 W laser power, 77 scanning cycles, and 39 μm face width. The fabricated V-shaped grooves showed excellent dimensional consistency, minimal heat-affected zone, and high contour fidelity. SEM characterization further confirmed that the groove inner wall exhibited a smooth surface with uniform granular microstructures and no obvious cracks or defects, indicating that femtosecond laser processing can achieve high-quality fabrication of drag-reduction microstructures on Ti6Al4V surfaces.

Overall, this study establishes an integrated design–simulation–fabrication framework for biomimetic drag-reduction functional surfaces on Ti6Al4V, providing both theoretical guidance for drag-reduction mechanism design and an effective technological route for the precision manufacturing of high-performance aerodynamic functional metallic surfaces.

## Figures and Tables

**Table 1:** Characteristic parameters of air.

Medium	Density (ρ, kg/m^3^)	Dynamic Viscosity (μ, N·s/m^2^)	Kinematic Viscosity (*v*, m/s^2^)
Air	1.225	1.789 × 10^−5^	1.46 × 10^−5^

**Table 2:** Simulation parameter table.

Number	Groove Shape	Dimensionless Depth	Aspect Ratio
1	V-shaped/U-shaped/Rectangle	10	0.5
2		15	1
3		20	1.5
4		25	2

**Table 3:** Selection table of computational domain boundary conditions.

Boundary Name	Location	Boundary Conditions
Inlet	Entrance	Velocity-inlet
Out	exit	Pressure outlet
Side	sides	Symmetry
Up	Top surface	Wall
Down	Bottom surface	Wall

**Table 4:** Theoretical and simulated values of surface friction coefficients in different regions.

Location (m)	Theoretical Value	Simulation Value	Absolute Error Value (%)
0.05	0.006645	0.006464	2.72
0.10	0.005785	0.005733	0.90
0.15	0.005334	0.005293	0.77
0.20	0.005036	0.005056	0.40
0.25	0.004816	0.004886	1.45

**Table 5:** Simulation results of V-shaped groove drag-reduction rate.

Number	h/µm	Aspect Ratio	Drag-Reduction Rate
1	90	0.5	8.68
2	90	1	9.32
3	90	1.5	7.75
4	90	2	2.27
5	72	0.5	8.95
6	72	1	8.87
7	72	1.5	5.87
8	72	2	3.55
9	54	0.5	10.70
10	54	1	13.10
11	54	1.5	7.93
12	54	2	5.11
13	36	0.5	10.21
14	36	1	12.10
15	36	1.5	12.00
16	36	2	6.63

**Table 6:** Simulation results of drag-reduction rate in U-shaped grooves.

Number	h/µm	Aspect Ratio	Drag-Reduction Rate
1	90	0.5	6.24
2	90	1	9.69
3	90	1.5	5.60
4	90	2	2.70
5	72	0.5	5.97
6	72	1	10.82
7	72	1.5	8.32
8	72	2	5.32
9	54	0.5	5.23
10	54	1	10.99
11	54	1.5	10.96
12	54	2	8.23
13	36	0.5	4.04
14	36	1	9.51
15	36	1.5	11.39
16	36	2	10.91

**Table 7:** Simulation results of drag-reduction rate of rectangular grooves.

Number	h/µm	Aspect Ratio	Drag-Reduction Rate
1	90	0.5	0.24
2	90	1	4.07
3	90	1.5	1.86
4	90	2	0.44
5	72	0.5	0.60
6	72	1	4.61
7	72	1.5	4.85
8	72	2	2.51
9	54	0.5	1.01
10	54	1	5.07
11	54	1.5	7.74
12	54	2	7.24
13	36	0.5	0.75
14	36	1	4.80
15	36	1.5	10.08
16	36	2	9.72

**Table 8:** Single-factor experimental protocol and results.

Number	Power (W)	Number of Scans	Face Width (µm)	Gutter Width (µm)	Depth of the Groove (µm)
1	0.5	70	40	56.7	45.1
2	1.0	70	40	57.4	53.8
3	1.5	70	40	59.8	60.0
4	2.0	70	40	63.3	68.1
5	1.0	30	40	57.6	24.6
6	1.0	50	40	57.0	42.7
7	1.0	70	40	57.4	53.8
8	1.0	90	40	57.3	72.8
9	1.0	70	25	39.9	50.6
10	1.0	70	30	46.8	52.5
11	1.0	70	40	57.4	53.8
12	1.0	70	50	64.2	57.2

**Table 9:** Factor level table of response surface method.

Level	Power (W)	Number of Scans	Face Width (µm)
−1	0.5	50	30
0	1.0	70	40
1	1.5	90	50

**Table 10:** Analysis of the regression results of groove width.

Project	Sum of Squares	Degree of Freedom	Mean Square	F Value	*p* Value	Significance
Model	660.03	9	73.34	220.37	<0.0001	Significant
A-Power	15.96	1	15.96	47.96	0.0002	
B-Number	2.00	1	2.00	6.01	0.0440	
C-Face width	635.46	1	635.46	1909.52	<0.0001	
AB	0.0625	1	0.0625	0.1878	0.6778	
AC	0.1600	1	0.1600	0.4808	0.5104	
BC	0.0025	1	0.0025	0.0075	0.9334	
A^2^	0.7427	1	0.7427	2.23	0.1788	
B^2^	0.3664	1	0.3664	1.10	0.3289	
C^2^	4.82	1	4.82	14.49	0.0067	
Residual	2.33	7	0.3328			
Misfitting item	0.3575	3	0.1192	0.2417	0.8635	Not significant
Pure error	1.97	4	0.4930			
Total sum	662.36	16				
*R*^2^ = 0.9965, $R_{adj}^{2}$ = 0.9920, $R_{pre}^{2}$ = 0.9867, C.V. = 1.03%						

**Table 11:** Analysis of trench depth regression results.

Project	Sum of Squares	Degree of Freedom	Mean Square	F Value	*p* Value	Significance
Model	2789.23	9	309.91	34.12	<0.0001	Significant
A-Power	478.95	1	478.95	52.73	0.0002	
B-Number	2122.26	1	2122.26	233.64	<0.0001	
C-Face width	79.38	1	79.38	8.74	0.0212	
AB	30.25	1	30.25	3.33	0.1108	
AC	0.0225	1	0.0225	0.0025	0.9617	
BC	0.9025	1	0.9025	0.0994	0.7618	
A^2^	21.13	1	21.13	2.33	0.1711	
B^2^	38.15	1	38.15	4.20	0.0796	
C^2^	20.10	1	20.10	2.21	0.1805	
Residual	63.59	7	9.08			
Misfitting item	26.12	3	8.71	0.9294	0.5041	Not significant
Pure error	37.47	4	9.37			
Total sum	2852.82	16				
	*R*^2^ = 0.9777, $R_{adj}^{2}$ = 0.9491, $R_{pre}^{2}$ = 0.8830, C.V. = 5.62%					

**Table 12:** Experimental scheme for verification of regression prediction model.

Number	Power (W)	Number of Scans	Face Width (µm)
1	0.5	60	40
2	0.7	70	38
3	0.7	80	38
4	1.0	80	40
5	1.5	60	40

**Table 13:** Optimal combination of process parameters.

Objective	Power (W)	Number of Scans	Face Width (µm)	Optimization Results (µm)
Minimum slot width	0.51	57	30	34.2
Maximum slot width	1.49	66	50	66.7
Minimum slot depth	0.50	50	34	31.0
Maximum slot depth	1.48	90	46	82.4
(54 µm, 54 µm)	0.77	77	39	/

## Data Availability

The original contributions presented in this study are included in the article. Further inquiries can be directed to the corresponding author.

## Abbreviations

The following abbreviations are used in this manuscript:

RSM	Response Surface Methodology
CFD	Computational Fluid Dynamics
RA	Aspect ratio
ANOVA	Analysis of variance
DNS	Direct Numerical Simulation
RANS	Reynolds Averaged Navier-Stokes
LES	Large Eddy Simulation

## References

[1] Zhang H.M., Yu W.J., Xu D.Q., Qin F., An J., Liu W., Yuan J., Zhu S. (2026). Active and passive drag reduction methods for supersonic internal flow paths in combined cycle engines. Acta Astronaut..

[2] Luo Y. (2015). Recent Progress in Exploring Drag Reduction Mechanism of Real Sharkskin Surface: A Review. J. Mech. Med. Biol..

[3] Graybill M.T., Xu N.W. (2024). Experimental Studies of Bioinspired Shark Denticles for Drag Reduction. Integr. Comp. Biol..

[4] Wang Y.Q., Wei Y.J., Weng D., Wang J. (2023). Aerodynamic Drag Reduction by the Trapezoid Spanwise Groove Inspired by Pigeon Feathers. Energies.

[5] Liu J.P., Xu J., Ding C.G., Shan D., Guo B. (2026). Numerical Investigation on Drag Reduction Mechanisms of Biomimetic Microstructure Surfaces. Biomimetics.

[6] Chen Q.H., Zhang C.Q., Cai Y.K., Luo X., Wang B., Song Q., Liu Z. (2023). Periodically oriented superhydrophobic microstructures prepared by laser ablation-chemical etching process for drag reduction. Appl. Surf. Sci..

[7] Bixler G.D., Bhushan B. (2013). Shark skin inspired low-drag microstructured surfaces in closed channel flow. J. Colloid Interface Sci..

[8] Martin S., Bhushan B. (2016). Fluid flow analysis of continuous and segmented riblet structures. RSC Adv..

[9] Khader M.A., Sayma A.I. (2022). Drag reduction within radial turbine rotor passages using riblets. Proc. Inst. Mech. Eng. Part E J. Process Mech. Eng..

[10] Liu Y., Wang H.M., Zhang Z.Y., Wang Y., Lu J., Zhu H., Xu K., Wang J., Zhang H., Lu H. (2024). Laser-liquid composite micromachining technologies: A review of research progress. J. Adv. Manuf. Sci. Technol..

[11] Li S., Zhang K., Liu W.J., Chen T., Li Q., Wang W., Bian H. (2025). Functional Properties of Micro–Nano Structure Surfaces Prepared by Femtosecond Laser: A Review. Adv. Eng. Mater..

[12] Zhang L., Wu T.C., Luo Y.F. (2025). Study on laser ablation of TC4 for drag reduction groove considering plasma shielding effect. Opt. Laser Technol..

[13] Xu Z., Liu R., Wang T.H., Chi Z.D., Wang Z.B., Li L. (2024). Simulation and fabrication of bionic sharkskin composite micro-nano wind resistance reduction structure. J. Exp. Fluid Mech..

[14] Wang Z., Zhao Q.Z., Wang C.W., Zhang Y. (2015). Modulation of dry tribological property of stainless steel by femtosecond laser surface texturing. Appl. Phys. A.

[15] Cheng H., Zhou F., Fei Z.H. (2023). Dry Friction Properties of Friction Subsets and Angle Related to Surface Texture of Cemented Carbide by Femtosecond Laser Surface Texturing. Coatings.

[16] Klebanoff P.S. (1954). Characteristics of Turbulence in a Boundary Layer with Zero Pressure Gradient.

[17] Robinson S. (1991). Coherent motions in the turbulent boundary layer. Annu. Rev. Fluid Mech..

[18] Moin P., Kim J. (1982). Numerical investigation of turbulent channel flow. J. Fluid Mech..

[19] Huai X., Joslin R.D., Piomelli U. (1997). Large-eddy simulation of transition to turbulence in boundary layers. Theor. Comput. Fluid Dyn..

